# Neonatal total gastrectomy as treatment for gastric rupture: Case report

**DOI:** 10.1016/j.ijscr.2025.111217

**Published:** 2025-03-27

**Authors:** Arturo Javier Cavazos Castro, Castro Anaya Harold, José Asz Sigall, María Elena Ortega Ramírez, Claudia Elitania Espinosa Guerrero, Horacio G. Carvajal

**Affiliations:** a Department of Pediatric Surgery, National Institute of Pediatrics, Av. Insurgentes Sur No. 3700-C, 04530 Mexico City, Mexico; b Department of Neonatology, National Institute of Pediatrics, Av. Insurgentes Sur No. 3700-C, 04530 Mexico City, Mexico; c Division of Pediatric Cardiothoracic Surgery, University of Utah, United States of America

**Keywords:** Gastrectomy, Neonatal gastric perforation, Pouchless esophagojejunostomy

## Abstract

**Introduction:**

Neonatal gastric perforation is an uncommon but life-threatening condition, rarely requiring gastrectomy. We report the case of a neonate requiring total gastrectomy secondary to suspected barotrauma leading to gastric perforation.

**Case presentation:**

A 2-day-old term male was referred to our institution in extremis following attempted resuscitation with makeshift positive airway pressure ventilation in the setting of respiratory distress. Exam was notable for a distended, peritonitic abdomen, and abdominal radiograph showed massive pneumoperitoneum. Exploratory laparotomy revealed an extensive anterior gastric perforation extending from the pylorus to the esophagogastric junction, along with gross ischemia of the posterior gastric wall. The patient underwent gastrectomy with pouchless retrocolic Roux-en-Y esophagojejunostomy reconstruction. He was kept NPO (nil per os) on total parenteral nutrition for seven days. Esophagram on postoperative day 7 demonstrated patency of the esophagojejunal anastomosis without leaks, and he was transitioned to formula via nasojejunal tube feeds supplemented with vitamins and pancreatic enzymes. He was discharged home on postoperative day 45. At latest follow-up 2.9 years after surgery, his height and weight were in the 10th percentile for his age.

**Clinical discussion:**

This report showcases the successful management of an extensive gastric perforation with gastrectomy and pouchless Roux-en-Y esophagojejunostomy. Multidisciplinary postoperative and outpatient care was essential to ensure a positive outcome.

**Conclusion:**

Neonatal gastric perforation is a rare condition with high morbidity and mortality, particularly in those born prematurely or low birthweight. This patient suffered from an extensive gastric perforation secondary to suspected barotrauma, undergoing gastrectomy and esophagojejunostomy without a pouch, achieving adequate nutritional status for his age.

## Highlights

• Neonatal gastric perforation is a rare life-threatening condition.
• Total gastrectomy in neonates is extremely rare.
• Most of these reports performed a pouch esophagojejunostomy.
• Risk factors are barotrauma, prematurity, low weight and gastric ischemia.
• Patients with total gastrectomy are at an increased risk of severe nutritional deficiencies.

## 1 Introduction

Neonatal gastric perforation is a rare but life-threatening entity, with a reported incidence of 1 in 5000 births and a mortality as high as 75 % [1,2]. Gastrectomy in the neonatal period is similarly extremely rare, with just a few isolated case reports. The majority of these reports performed a Hunt-Lawrence pouch esophagojejunostomy with the aim of providing a reservoir which theoretically diminishes the risk of dumping syndrome and improves postoperative weight gain [2,3].

We present the case of a 2-day-old boy transferred to our institution with an extensive gastric perforation, presumably related to barotrauma, which required gastrectomy with a pouchless Roux-en-Y esophagojejunostomy reconstruction, achieving adequate postoperative nutrition.

This manuscript was prepared following the SCARE guidelines [4].

## 2 Case report

A 37-week term boy with adequate birth weight was born by cesarean section at a small outside hospital. At birth, with a weight of 3300 g, height of 50 cm. Despite initial APGAR score of 8/9 and Silverman-Andersen score of 2, he developed respiratory distress requiring supplemental oxygen. He continued to present worsening respiratory distress, prompting escalation with an artisanal hospital-made bubble continuous positive airway (CPAP) support system with a reported rate of 6 l/min and a positive pressure of 4 cmH_2_O. After two days of persistent respiratory distress despite positive pressure ventilation, he was transferred to our institution.

**Fig. 1:**
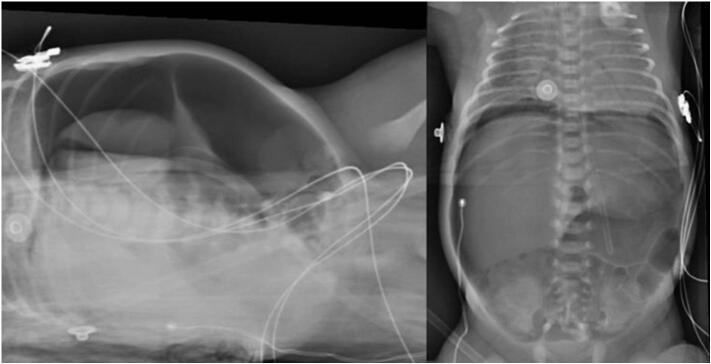
Anteroposterior and Right lateral decubitus abdominal radiograph showing pneumoperitoneum.

**Fig. 2:**
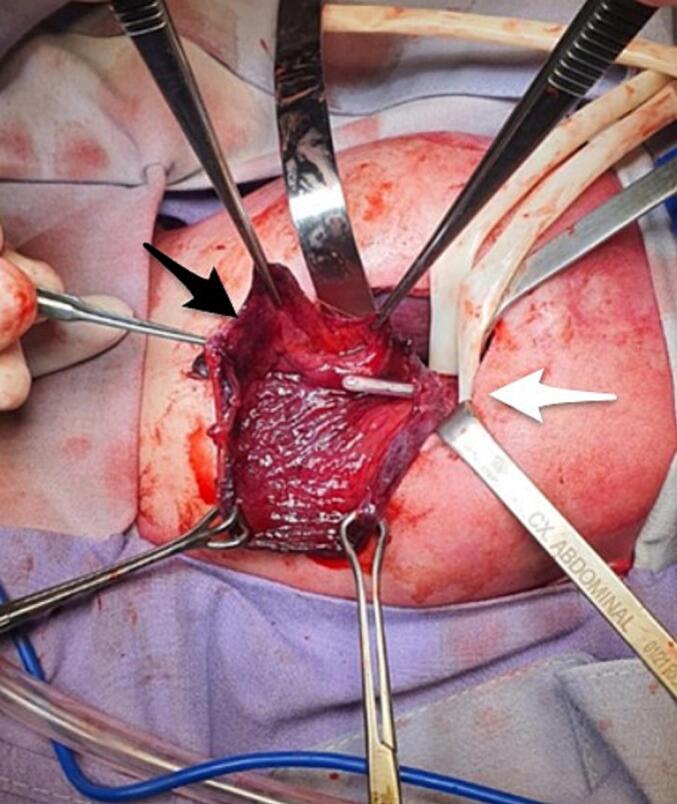
Extensive anterior gastric perforation with necrotic borders and posterior gastric wall precluding primary repair. Black arrow shows the esophagogastric junction with NG tube. White arrow shows the pylorus.

**Fig. 3:**
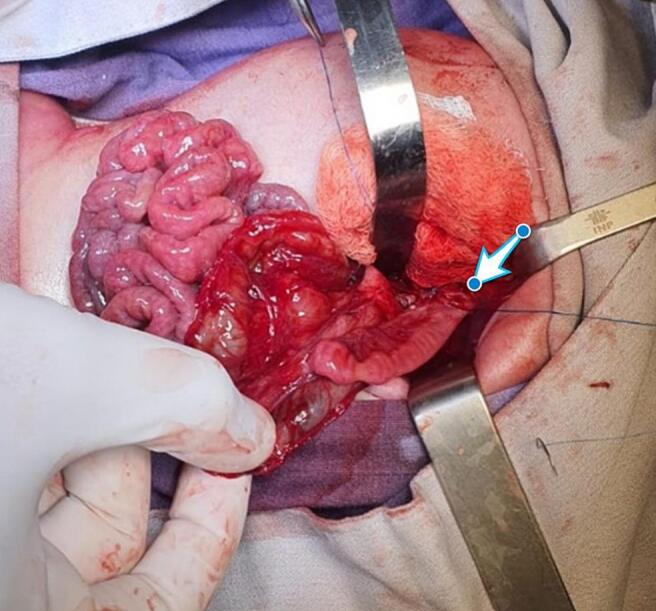
Retrocolic pouchless Roux-en-Y esophagojejunostomy pointed by white arrow.

On arrival to the neonatal intensive care unit (NICU), the patient was hemodynamically unstable on pressor support. Physical exam was notable for respiratory distress and a distended, peritonitic abdomen. Laboratory tests showed hemoglobin 12 mg/dl, hematocrit 36 %, WBC 11.4 × 10^9^/l, and platelets 295 × 10^9^/l. Abdominal radiographs showed massive pneumoperitoneum (Fig. 1), meriting an urgent laparotomy. Intraoperative findings were notable for an anterior gastric perforation extending from the gastroesophageal junction to the pylorus, along with extensive ischemia of the posterior gastric wall (Fig. 2). He remained on epinephrine, milrinone, and vasopressin intraoperatively, with transfusion of 1 unit of packed red blood cells at 20 ml/kg. Given his surgical findings and hemodynamic instability, we decided to proceed with a total gastrectomy and retrocolic Roux-en-Y pouchless end-to-end esophagojejunostomy (Fig. 3). We created the esophagojejunal anastomosis using a single layer of 5–0 polypropylene interrupted stitches, and the jejunojejunal anastomosis with a single layer of 6–0 polyglycolic acid interrupted stitches. Analysis of the explanted stomach by pathology reported a complete loss of architecture of the anterior gastric wall due to a large perforation. On the posterior surface, a 1.5 × 1.5 cm perforation was identified, with irregular and friable edges as well as surrounding tissue ischemia.

**Fig. 4:**
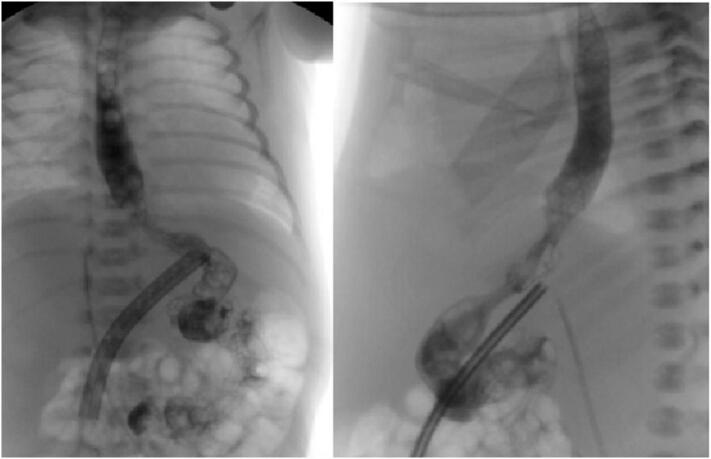
Anteroposterior and left lateral esophagogram, visualizing adequate passage of contrast medium through the pouchless esophagojejunal anastomosis.

Following surgery, the patient was managed by a multidisciplinary team comprised of neonatologists, gastroenterologists, nutritionists, and pediatric surgeons. He returned to the NICU on milrinone, epinephrine, and norepinephrine support, which were slowly weaned over the following 3 days. He was kept NPO and supported on total parenteral nutrition with SMOFLipid for the first week after surgery. On postoperative day (POD) 7, he underwent an esophagram which showed adequate patency of the esophagojejunostomy with no contrast leak (Fig. 4). He subsequently started on a hypercaloric elemental formula via nasojejunal tube, which was gradually increased to a goal of 180 ml/kg/day and supplemented with vitamin B12, vitamin D, pancreatic enzymes, ferrous sulfate, and calcium.

Following an uneventful postoperative course, the patient was discharged home on POD 45. Over the past 2 years and 9 months, he has been regularly followed in clinic by pediatric surgery, neonatology, and general pediatrics. His current nutritional regimen is comprised of a normal diet supplemented with elemental formula (32 oz per day), vitamin D (800 IU), zinc (30 mg), ferrous sulfate (60 mg), and pancreatic enzymes (100 mg per meal). Additionally, he receives vitamin B12 (1000 μg) injections monthly. At his most recent follow-up visit, the patient weighed 11.6 kg (10th percentile), with a height of 89 cm (9th percentile) and a weight-for-height ratio of 11.8 %. His latest laboratory exams reported a hemoglobin of 13 g/dl, hematocrit 39 %, serum protein 6.8 g/dl, albumin 4.6 g/dl, prealbumin 21 mg/dl, transferrin 331 mg/dl, vitamin D levels 41 ng/ml, vitamin B12 levels 84 pg/dl, and iron 84 μg/dl.

## 3 Discussion

The current case report highlights the importance of prompt surgical treatment and multidisciplinary postoperative care in the case of suspected neonatal gastric perforation. The suspected etiology of this patient's gastric perforation was barotrauma secondary to the use of an artisanal bubble CPAP for respiratory support. Although the extent of his injury and resulting tissue necrosis precluded primary repair of the gastric perforation, he experienced a relatively benign postoperative course following total gastrectomy with pouchless Roux-en-Y esophagojejunostomy. Multidisciplinary care by a team comprised of neonatologists, gastroenterologists, nutritionists, and pediatric surgeons was essential for adequately resuscitating this patient and optimizing his nutritional support.

Spontaneous neonatal gastric perforation is a rare, life-threatening condition with a reported incidence of 1:5000 live births and a mortality rate ranging from 9 to 68 % [1,[5], [6], [7]]. Gastric perforations are observed in male neonates four times more often compared to females [5,8], and both prematurity and low birth weight have been associated with greater risk of perforation and associated mortality [1,2,[5], [6], [7],^9^]. It usually occurs between the second and seventh days of age [1,2,6]. The majority of cases are idiopathic, accounting for 47 % of cases in a systematic review of 51 articles on neonatal gastric perforation [1]. The most cause for non-idiopathic gastric perforation is iatrogenic, including secondary to barotrauma as suspected in our patient, followed by underlying gastrointestinal pathologies, such as necrotizing enterocolitis, small bowel obstruction, esophageal atresia or tracheoesophageal fistula [1].

The pathophysiology behind the increased risk of gastric perforation in prematurity and low birth weight is thought to be due to defects of the gastric muscle walls, including immature tissues, and a lack of intestinal pacemaker cells and C-kit mast cells [1,10]. Significant gastric distension, as seen with positive pressure ventilation, can cause mucosal ischemia, necrosis, and resulting perforation. Gastric ischemia can also be caused by hypotension, hypoxia, vasculopathy, thrombosis, and congenital heart disease. Similarly, medications such as corticosteroids, indomethacin, or ibuprofen can injure gastric mucosa, potentially resulting in perforation. Immature gastric peristalsis, frequently present at birth, favors an increase in gastric pressures, while gastric atony and pyloric spasm may lead to punctiform perforations of the gastric wall [2,4,11,12]. In the case of our patient, the suspected mechanism behind the extensive gastric ischemia and perforation was the use of the artisanal hospital-made bubble CPAP.

Most neonates with gastric perforation present with abrupt abdominal distension and nonspecific symptoms, such as lethargy, fever, intolerance to feeds, and respiratory distress. Physical exam is limited to abdominal induration and pain on abdominal palpation [5,10]. Massive pneumoperitoneum on abdominal radiographs, suggestive of gastric perforation, is a commonly seen imaging finding in these patients [7]. The location of the gastric perforation can vary, though most are found on the greater curvature (37–73 %) and the anterior wall [5,7]. Likewise, the size of the perforation can vary widely, although those measuring 1.5 cm or greater have been associated with increased mortality [3,5,6,13].

Initial conservative treatment with peritoneal drainage may be useful in critically unstable neonates, although surgery is almost always required. Primary repair of the perforation is preferred in patients with punctiform perforations, while those with larger perforations may require debridement of any necrotic tissue and gastrorrhaphy or partial gastrectomy [6,7]. There are few reported cases of neonatal perforation requiring a total gastrectomy [2,3]. The options for reconstruction after gastrectomy in neonates are mostly extrapolated from the adult gastric cancer population, and are classified according to whether they preserve the continuity of the duodenum or not [14,15].Preserving duodenal continuity is ideal, as it regulates intestinal motility and stimulates many cells with hormonal, paracrine and neurocrine effects that help in the digestion of the food. Duodenal preservation may be accomplished by a Roux-en-Y double-tract esophagojejunostomy or an esophagojejunostomy with functional jejunal interposition (Longmire procedure) [14,15]. Among the few reported cases of neonates who underwent gastrectomy, several repair techniques, including colonic interposition, delayed Hunt-Lawrence pouch, and jejunostomy, have been described [2,3,15].

When choosing a reconstructive procedure without duodenal passage, the most important consideration is to prevent biliary reflux into the esophagus. This is accomplished by a Roux-en-Y esophagojejunostomy, which may or may not be accompanied by the creation of a jejunal pouch. The jejunal pouch was designed to replace the stomach and increase the reservoir capacity of the jejunal substituent (Hunt-Lawrence procedure), decreasing the risk of dumping syndrome and biliary regurgitation while facilitating digestion [[16], [17], [18]]. The creation of a jejunal pouch, however, is not risk-free, as ischemia may occur in the upper acute angle when rotating the jejunum to create the pouch or in the case of a shortened jejunum or mesentery [16,18]. In the case of our patient, a gastrectomy was warranted due to the extensive anterior gastric perforation, surrounding tissue necrosis, and posterior wall ischemia. Given the small size of the unused jejunum and his intraoperative hemodynamic lability, we not to construct a jejunal pouch for the esophagojejunostomy. Gastrectomy should be avoided when possible to prevent nutritional deficiencies, dumping syndrome, reflux esophagitis, and failure to thrive. The pouchless esophagojejunal reconstruction can be a viable surgical strategy when pouch creation is not feasible or is technically challenging due to factors such as hemodynamic instability or a very small intestine. In such cases, a pouchless reconstruction can be a viable surgical option, leading to good outcomes, including adequate growth and nutritional status thanks to a multidisciplinary approach.

Patients who undergo gastrectomy are at an increased risk of severe nutritional deficiencies and failure to thrive. Gastrectomy results in the loss of digestive enzymes, reduced stimulation of pancreatic and biliary secretions, inappropriate mixing of food with pancreatic enzymes and bile, increased bowel motility, and excessive bacterial colonization. These patients may also develop anemia, primarily due to the malabsorption of several nutrients. Microcytic anemia may result from iron malabsorption, as the absence of gastric acid impedes the reduction of ferric iron (Fe^3+^) to ferrous iron (Fe^2+^) which is more readily absorbed in the duodenum. Conversely, macrocytic anemia stems from the absence of parietal cells, found mainly in the fundus and body of the stomach, which leads to intrinsic factor deficiency and consequently malabsorption of vitamin B12 in the ileum. The lack of gastric acid favors bacterial overgrowth, which damages the gastrointestinal epithelium and deconjugates bile acids, leading to fat malabsorption. Meanwhile, accelerated gastrointestinal transit reduces calcium absorption, and the presence of steatorrhea produces insoluble calcium soaps, leading to bone metabolism disorders. Accelerated transit also alters the glucose uptake, as well as the absorption of lipids and proteins [14,16].

To reduce the risk of these complications, our patient was managed by a multidisciplinary team composed of neonatologists, gastroenterologists, nutritionists, and pediatric surgeons. He was fed with a hypercaloric formula supplemented with vitamins B12 and D, pancreatic enzymes, ferrous sulfate, and calcium during the postoperative period, and has been followed in each specialty's respective outpatient clinic. Thanks to this multidisciplinary approach, the patient has been able to achieve an adequate nutritional status for his age without nutritional deficiencies.

## 4 Conclusion

Neonatal gastric perforation is a rare and severe condition with high morbidity and mortality, particularly in preterm and low-birthweight infants. To our knowledge, this case represents the first reported neonatal total gastrectomy due to suspected barotrauma reconstructed with a pouchless Roux-en-Y esophagojejunostomy. Despite the challenges, multidisciplinary postoperative care enabled a successful recovery with adequate growth and nutrition. Our experience suggests that pouchless esophagojejunal reconstruction can be a viable option leading to good outcomes when pouch creation is not feasible or technically challenging due to anatomic factors or hemodynamic instability.

## CRediT authorship contribution statement

Harold Castro Anaya– Data collection and analysis
Arturo Javier Cavazos Castro – Data collection, analysis and writing paper
María Elena Ortega Ramírez– Study concept
José Asz Sigall – Study concept, interpretation
Claudia Elitania Espinosa Guerrero – Data collection and analysis
Horacio G. Carvajal – Analysis and writing paper.

## Informed consent

Parental consent for minors: Written informed consent was obtained from the patient's parents for publication and any accompanying images.

## Ethical approval

This case report is exempt from ethical approval by our institution. The ethics committee at our institution, does not review case reports that do not involve prospective intervention or significant risk to the patient. Due to this case report is a retrospective, single case with anonymized data, it was not considered to be reviewed.

## Guarantor

Arturo Javier Cavazos Castro is the guarantor of the work.

## Authorship

All authors attest that they meet the current ICMJE criteria for Authorship.

## Funding

This research did not receive any specific grants from funding agencies in the public, commercial, or non-profit sectors.

## Declaration of competing interest

The authors declare that they have no competing economic interests or known personal relationships that could have influenced the work reported in this document.
